# Stress and the menopausal transition in Campeche, Mexico

**DOI:** 10.1186/s40695-018-0038-x

**Published:** 2018-06-18

**Authors:** Lynnette Leidy Sievert, Laura Huicochea-Gómez, Diana Cahuich-Campos, Dana-Lynn Ko’omoa-Lange, Daniel E. Brown

**Affiliations:** 1 Department of Anthropology, Machmer Hall, 240 Hicks Way, UMass Amherst, Amherst, MA 01003-9278 USA; 2 Departamento de Sociedad y Cultura, El Colegio de la Frontera, ECOSUR, Campeche, México; 3 Department of Pharmaceutical Science, University of Hawai’i at Hilo, Hilo, HI USA; 4 Department of Anthropology, University of Hawai’i at Hilo, Hilo, HI USA

**Keywords:** Menopause, Stress, Hot flashes, Night sweats, Fatigue, Sleep difficulties, Depression

## Abstract

**Background:**

Stress has been implicated as a factor in the presence and severity of symptoms during the menopausal transition. Our primary aim was to test the hypothesis that stress-sensitive biological measures and self-reported stress would be positively associated with a greater likelihood and intensity of hot flashes. Our secondary aim was to examine measures of stress in relation to the most often reported symptoms in Campeche, Mexico. We also hypothesized ethnic differences (Maya versus non-Maya) in relation to measures of stress and symptom reports.

**Methods:**

Participants aged 40–60 (*n* = 305) were drawn from multiple sites across the city of San Francisco de Campeche to achieve a generally representative sample. Measures included C-reactive protein (CRP), an indicator of inflammation; Epstein-Barr virus antibodies (EBV-Ab), an indicator of immune function; the Perceived Stress Scale (PSS); a symptom checklist; anthropometric measures; and a questionnaire that elicited symptoms, ethnicity (based on language, birthplace, and last names of the woman, her parents, and her grandparents) and ten dimensions of socioeconomic status (SES). The relationships between symptoms and stress-sensitive biological and self-reported measures were examined in bivariate analyses, and with logistic and linear regressions.

**Results:**

The twelve most common symptoms reported, in descending order of frequency, were tiredness, muscle and joint pain, nervous tension, problems concentrating, feeling depressed, difficulty sleeping, headaches, feeling of ants crawling on the skin, loss of interest in sex, urinary stress incontinence, hot flashes, and night sweats. PSS scores were significantly associated with the likelihood of seven symptoms (yes/no), and with the intensity of ten symptoms after controlling for ethnicity, SES, education, cohabitation status, parity, smoking, body mass index, and menopausal status. The stress-sensitive biological measures of immune function (EBV-Ab and CRP) were not significantly associated with midlife symptoms. The PSS was associated with more symptoms among the Maya (e.g., feeling nervous/tense and having difficulty concentrating) than non-Maya.

**Conclusion:**

PSS scores were associated with the intensity, but not the likelihood, of hot flashes. Other symptoms were also associated with self-reported stress but not with physiological measures. Maya/non-Maya differences may indicate that either symptoms or stress were experienced and/or reported in culture-specific ways.

## Background

The menopausal transition is often characterized by hot flashes and night sweats [1], fatigue and body aches [2], difficulty sleeping [3], and transient depression [4]. Some symptoms can be attributed to the changing hormone levels associated with the loss of ovarian follicles, including fluctuating estradiol and increases in follicle stimulating hormones [5, 6]. However, some symptoms may be better explained by combining physiological information with the social changes that coincide with this time of life. For example, a woman’s children are likely to be adolescents with their own challenges, husbands may be undergoing transition in social status such as retirement or struggling with health issues, and parents may be in need of substantial levels of care [7, 8].

During the menopausal transition, stress may be a contributor to trouble sleeping, depression [9, 10], and/or symptoms that may have a psychosomatic component [11]. For example, in cross-cultural work among women aged 45–55, Sievert et al. [12] found that *job* change was associated with an increased likelihood of nervous tension, difficulty concentrating, headaches, and fatigue in the U.S., but not in Spain. In Spain, but not the U.S., *household* change was associated with depressed mood and difficulty concentrating. These differences show that stress is variable and context dependent. It appears that job change may be experienced as more stressful in the U.S., whereas household change may be more stressful in Spain.

Specific to hot flashes, stress has been identified as a determinant in some [13–17], but not all [12, 18, 19] studies of factors associated with hot flashes. In a laboratory setting, where symptomatic women were exposed to a variety of stressors, there were 57% more self-reported hot flashes during stress periods compared to non-stress periods [20]. In the Study of Women’s Health Across the Nation (SWAN), after adjusting for ethnicity, lifestyle, and other confounding variables, self-reported perceived stress was significantly associated with self-reported vasomotor symptoms (adjusted odds ratio 1.4, 95% confidence interval 1.2–1.6) [15], and significantly related to a longer persistence of self-reported hot flashes into the postmenopausal period [13]. In a 13-year longitudinal study in Philadelphia, women who reported moderate or severe hot flashes during the study period had a higher baseline Perceived Stress Scale (PSS) score (21.9) compared to women with mild hot flashes (19.5) or no hot flashes (18.2, *p* < 0.01). Stress was not significantly associated with the duration of self-reported hot flushes in a multivariable model [14].

Cortisol is a stress-sensitive biological measure [21] that has been examined in relation to hot flashes. Two early laboratory studies showed an increase in cortisol levels during and after monitored hot flashes [22, 23]. In the Seattle Women’s Health Study, women with increased urinary cortisol had significantly greater self-reported hot flash and cold sweat symptom severity compared to women without increased cortisol [24]. In Modena, Italy, women with self-reported severe hot flashes had significantly higher levels of 24-h urinary cortisol compared to women with none to moderate vasomotor symptoms [25]. Hot flash report has also been associated with higher salivary cortisol levels in the early afternoon [26]. In a small study where women with hot flashes were measured by an ambulatory monitor, objectively measured hot flashes were associated with significantly higher salivary cortisol levels at 15, 30, and 45 min post-waking compared to women without biometrically measured hot flashes [27].

Not all studies have shown a consistently positive relationship between hot flashes and cortisol levels. For example, hot flash report has not been associated with the cortisol awakening response or diurnal variation in cortisol levels [26, 28, 29]. One study found greater self-reported hot flash severity associated with a flatter diurnal slope in salivary cortisol [30].

Self-reported hot flashes and other symptoms have been shown to vary across ethnicity within the same country [31–33]. Self-reported stress has also been shown to vary with ethnicity. For example, Brown [34, 35] compared levels of stress across two Filipino-American ethnic groups to show that individuals from Visayan backgrounds self-reported significantly higher levels of stress compared to individuals of Ilocano descent. At the same time, there was no difference in the 24-h excretion rates of norepinephrine and epinephrine between the two groups. Brown also found that Filipino American women (mostly Ilocanos) were significantly more likely to record being anxious in a diary compared to European American women, but European Americans had higher elevations in ambulatory blood pressure when they did report anxiety [36]. Ethnic differences were also found in response to doing household chores: Filipino American women were more likely to report being anxious during chores than European Americans, but the European American women had higher diastolic BP while doing chores than the Filipino Americans [36]. Ethnic differences in the report of stress may reflect psychosocial differences [37], or culturally-based reporting biases [38]. For these reasons, the study reported here examined self-reported stress and symptom frequencies between Maya and non-Maya women.

Previous studies of menopause among Maya women in the Yucatán Peninsula of Mexico found an early mean recalled age at natural menopause of 44 years, compared to 52.5 years in the U.S. [39–41]. An in-depth ethnographic study documented an absence of self-reported hot flashes among rural Maya women [42]. According to Beyene, Maya women explained menopause as something that occurred when a woman used up her menstrual blood ([43], page 119). These women perceived menopause to be “a life stage free of taboos and restrictions, offering increased freedom of movement” (p.120). Other investigators recorded higher levels of hot flash frequencies among urban (49%) and rural (41%) Maya women in the Yucatán peninsula [44].

This study administered the PSS, as used in the SWAN and Philadelphia studies, to measure self-reported stress. To our knowledge, this will be the first study to examine hot flashes and other symptoms at midlife in relation to Epstein-Barr virus antibodies (EBV-Ab) [45, 46]. Both C-reactive protein (CRP) and EBV-Ab have been positively associated with high stress levels [47, 48]. CRP is an acute-phase protein that is commonly used as a measure of general inflammation. Because chronic stress is associated with elevated inflammation levels [49], this protein has been used as a marker of both acute and chronic stress [50, 51]. With regard to EBV-Ab, most people are chronically infected with EBV. When an individual is stressed, down-regulation of the immune system allows the virus to replicate, and antibodies to the virus increase in the blood stream. Accordingly, an elevated EBV-Ab level has been used as a biological marker of stress [47, 52].

The primary aim of this study was to test the hypothesis that two biological measures potentially sensitive to stress and a self-reported measure of stress would be associated with a higher likelihood and intensity of hot flashes after controlling for potential confounders. Our secondary aim was to examine the stress-sensitive measures and self-reported stress in relation to the most commonly reported symptoms in Campeche, Mexico. Based on the results of other cross-cultural studies [12, 36] detailed above, we paid particular attention to ethnic differences in nervous tension, difficulties concentrating, headaches, fatigue, and depressed mood, as well as hot flashes and trouble sleeping. We hypothesized that all stress measures would be associated with the frequency and intensity of each of the 12 most-reported symptoms in bivariate analyses, and after controlling for potential confounders. We also hypothesized ethnic differences (Maya vs. non-Maya) in relation to measures of stress and symptom reports [38]. Other variables that could affect both stress measures and symptoms were collected, including age, menopausal status, level of education, socioeconomic status (SES), body mass index (BMI), ethnicity, marital status and cohabitation with husband or partner, parity, and smoking habits.

## Methods

### Sample

The study took place in San Francisco de Campeche, a city of approximately 250,000 people [53] located on the western coast of the Yucatan peninsula. Nearly 12% of the city’s population speaks Maya [53]. Women aged 40–60 years were drawn from businesses, schools, the city market, and by presentations given in homes. The use of several recruitment methods assured a diverse, although not random, sample of the city’s population. These participants make up the urban component of a larger study of menopause in the state of Campeche [54]. In the city, a total of 305 women participated in interviews and anthropometric measures, with a subsample of 162 participants providing finger stick blood samples. Of those 162 women, 109 provided sufficient blood for the assay of both CRP and EBV-Ab levels.

The study was approved by the Institutional Review Board of the University of Massachusetts Amherst; the Human Subjects Committee of the University of Hawaii at Hilo; and the Committee for Ethics in Research of the Secretary of Health in the State of Campeche, Mexico. All participants signed a letter of consent after lengthy explanation in Spanish.

### Measures

All participants answered questions related to their age, education, parity, and smoking status. An SES index was created from 10 dimensions related to housing construction, household composition, and infrastructure, such as, access to drinking water and type of cooking fuel. Within the city of Campeche, the range in SES index was from 22 to 39. With regard to marital status, 96% of married women (*n* = 160) and 73% of women with a partner (*n* = 26; *union libre*) lived with their partner and, therefore, the variable of interest used in the analyses here was whether or not a woman cohabited with a husband or partner.

Maya/non-Maya ethnicity was assessed on the basis of each woman’s two last names, whether she could speak or understand Maya, and place of birth. The same information was collected with regard to her parents and grandparents. Women were categorized as Maya, not Maya, or not able to be clearly defined on the basis of this information from all three generations. There were 40 participants for whom an ethnic was unclear because of missing information (e.g., not everyone knew the language spoken by their grandparents).

Menopausal status was defined by STRAW+ 10 stages: (1) regular menstruation, (2) changes in the number of days or quantity of blood, (3) more or less frequent menstruation, (4) a change in periods of more than 6 days, (5) 2 months or more have passed without a period, and (6) more than 12 months have passed without a period [55]. Stages 1 and 2 were categorized as pre-menopausal, stages 3 to 5 as peri-menopausal, and stage 6 as postmenopausal.

Stature was measured with a Seca 213 stadiometer to the nearest 0.1 cm. Weight was measured to the nearest 0.1 kg with a digital scale. BMI was computed as kg/m^2^.

All participants completed the PSS that has been previously used in Mexican populations [56]. The PSS is a well validated 10-item questionnaire that directly queries levels of stress experienced in the past month, and the degree to which one’s life is unpredictable, uncontrollable, and overloaded [57, 58].

Participants were asked about the presence or absence of 19 symptoms during the past 2 weeks including hot flashes (*Ha tenido calores o bochornos*?) and night sweats **(***En la noche ha tenido sudoraciones*?). This “everyday symptom list” has been used in many studies [59–61], including in Mexico [62]. Symptom intensities were reported as: 0 = *nada*; 1 = *un poco*; 2 = *mucho*; and 3 = *muchisimo*. Twelve symptoms had a frequency of 45% or higher in the city of Campeche. The cut off of 45% was selected in order to include hot flashes and night sweats in the analyses below. The 12 symptom reports were totaled to derive a total number of symptoms reported for each individual. Also, the intensity of the 12 symptoms were totaled to derive a total symptom intensity score for each participant.

Blood was collected by finger stick onto Whatman #903 Protein Saver filter paper sample cards [47], dried for 4 h, and immediately frozen in the Huicochea laboratory at ECOSUR, Campeche. The cards were carried to the United States by LLS, and mailed overnight to the University of Hawaii at Hilo with ice packs. The cards were then transferred to freezer storage at − 30 °C until analysis.

To determine the presence of EBV – Viral Capsid Antigen (VCA) in dried blood spot samples, an EBV-VCA enzyme-linked immunosorbent assay (Diamedix Corporation, Miami Lakes, FL), was modified for sampling dried blood spots. Briefly, a sample of each blood spot was taken by punching a single 6 mm disc using a standard hand held hole puncher. The blood spot samples were incubated in elution buffer overnight, on a platform shaker at low speed. 100 uL of the cut-off calibrator, controls and samples were transferred to the antigen wells. The samples and controls were allowed to incubate at room temperature for 30 min. The contents of the wells were discarded, and the wells were washed three times with wash solution. 100 uL of conjugate was pipetted into each well, and allowed to incubate at room temperature for 30 min. The contents were discarded, and the wells were washed three times in wash solution. Next, 100 uL of the substrate was pipetted into each well, and the wells were incubated at room temperature for 30 min. After incubation with substrate, 100 uL of stop solution was pipetted into each well. The absorbance was determined at 450 nm. All controls and samples were assayed in duplicate [45].

To determine the index value for each participant, the following formula was used:

$$ \frac{\mathrm{Absorbance}\kern0.5em \mathrm{of}\kern0.5em \mathrm{sample}}{\begin{array}{l}\mathrm{Mean}\kern0.5em \mathrm{absorbance}\kern0.5em \mathrm{of}\\ {}\mathrm{cut}\hbox{-} \mathrm{off}\kern0.5em \mathrm{calibrator}\end{array}}=\mathrm{Index}\kern0.5em \mathrm{value} $$

Samples with an index value ≥1.10 were determined to be positive for VCA IgG antibody.

CRP enzyme-linked immunosorbent assay (Abcam, Cambridge, MA) was used to quantitatively measure human CRP in blood spots following the methods of McDade et al. [63]. CRP values in blood spots were converted into the equivalent values of CRP in plasma by the following: (CRP_bloodspot_ * 1.15) – 0.13 = CRP_Plasma_ [63]. None of the participants had a CRP_Plasma_ value greater than 10.0 mg/L, an indicator of an active infection which would have led to exclusion from analyses involving CRP and EBV-Ab [64].

### Analyses

PSS scores, EBV-Ab levels, and CRP_Plasma_ levels were appraised for normal distribution. PSS scores were normally distributed and examined in relation to ethnic categories (Maya, not Maya, difficult to categorize) by ANOVA and in relation to each symptom (yes/no) by t-tests. EBV-Ab and CRP_Plasma_ levels were not normally distributed, and therefore were examined in relation to ethnic categories and in relation to each symptom by two-tailed Mann Whitney tests. Spearman correlations were examined between EBV-Ab values, CRP_Plasma_ levels, and PSS scores.

Logistic regressions were performed with each of the 12 symptoms (none vs. any level of symptom experience) as a dependent variable in a separate regression model. Analyses were carried out separately for each of the three stress measures – PSS scores, EBV-Ab values, and CRP_Plasma_ levels; therefore, there were three analyses carried out for each of the 12 symptoms. BMI, SES, education, ethnicity, cohabiting with a husband or partner, parity, smoking, and menopausal status were covariates. Because of the correlation among the covariates SES and education (*r* = .465, *p* < 0.001), and in order to achieve the best set of variables associated with each symptom, backward stepwise regression was carried out with a probability for entry set at 0.05 and probability for removal set to 0.10. Because of the multiple testing, we applied an adjusted *p*-value of *p* ≤ 0.001 to determine significance. Logistic regressions were repeated separately for women categorized as Maya and non-Maya.

Linear regressions with backwards elimination were carried out for all participants with intensity of symptom reports (*nada, un poco, mucho, muchisimo*) as dependent variables and PSS scores, EBV-Ab values, CRP_Plasma_ levels, BMI, SES, education, ethnicity, cohabiting with a husband or partner, parity, smoking, and menopausal status as covariates. As described above, analyses for each symptom were carried out separately for the three stress variables, and analyses were repeated separately for women categorized as Maya and non-Maya, respectively.

## Results

**Fig. 1:**
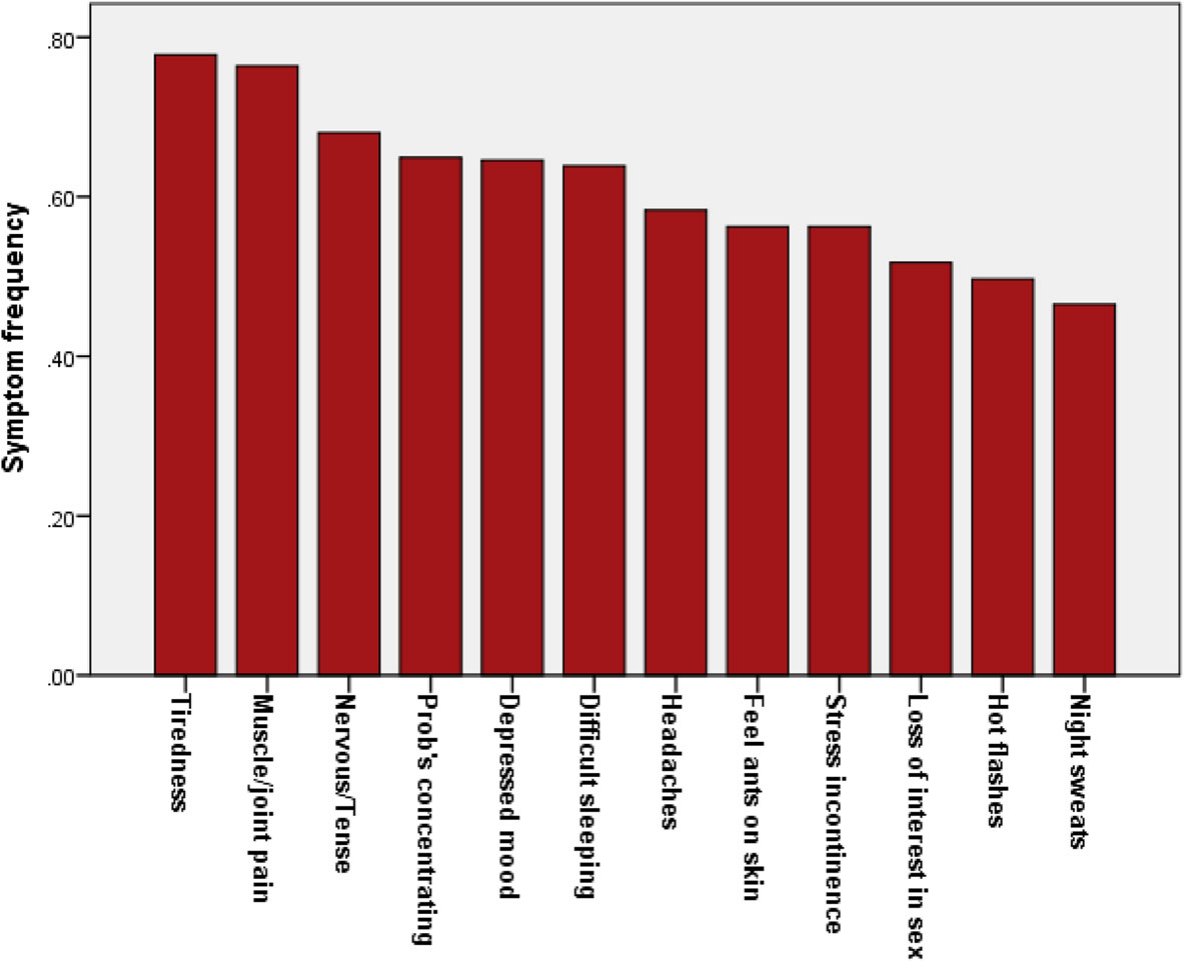
Frequency of the 12 most often reported symptoms among Maya and non-Maya women living in the city of Campeche, Mexico (*n* = 305)

Table 1 presents some characteristics of the sample by ethnicity. The Maya had a significantly lower SES index than non-Maya, but there were otherwise no significant ethnic differences in the listed characteristics. There were no significant differences in the PSS score between Maya and non-Maya women (*t* = 1.3, ns); Maya women had significantly higher EBV-Ab (two-tailed Mann Whitney test, *p* < 0.05), but there was no significant ethnic difference in CRP_Plasma_ levels. There were no significant ethnic differences in the frequency of symptoms, the total number of reported symptoms, or the total symptom intensity scores (two-tailed t-tests, ns). Figure 1 shows the frequency of reported symptoms for the entire sample.

**Table 1:** Participant information. Means ± standard deviations, numbers of participants, or percentages shown

	Maya	Non-Maya	Could not classify	All
N	144	121	40	305
Age at interview Mean ± s.d.	47.9 ± 5.0	46.9 ± 5.0	47.5 ± 5.0	47.5 ± 5.0
BMI (kg/m^2^) Mean ± s.d.	31.3 ± 5.2	30.3 ± 5.8	29.1 ± 5.3	30.6 ± 5.5
SES Index^a^ Range 22–39. Mean ± s.d.	32.8 ± 2.4	33.4 ± 2.3	33.4 ± 2.4	33.1 ± 2.4
Education (yrs) Mean ± s.d.	12.8 ± 4.4	13.8 ± 4.0	13.3 ± 4.4	13.2 ± 4.2
Menopause status (%)				
Pre-menopausal	40.3	47.9	42.5	43.6
Perimenopausal	20.8	24.0	22.5	22.3
Post – menopausal	38.9	28.1	35.0	34.1
% cohabiting with husband or partner	59.0	57.0	70.0	59.7
Parity Mean ± s.d.	2.0 ± 1.1	2.1 ± 1.2	2.0 ± 1.4	2.0 ± 1.2
Smoking (%)	10.4	14.9	12.5	12.5
PSS score Mean ± s.d. n = 305	1.55 ± 1.6	1.04 ± 0.6	1.58 ± 1.7	1.35 ± 1.3
EBV-Ab level* Mean ± s.d. *n* = 162	4.59 ± 1.4	4.06 ± 1.7	3.83 ± 1.6	4.30 ± 1.6
CRP_plasma_ level Mean ± s.d. *n* = 157	16.78 ± 5.2	17.65 ± 5.9	17.95 ± 4.5	17.28 ± 5.4
Total symptom score (range 0–12, based on 12 most common symptoms)	7.4 ± 3.0	7.4 ± 2.7	6.9 ± 3.1	7.3 ± 2.9
Total symptom intensity score (range 0–33, based on 12 most common symptoms)	10.9 ± 6.4	10.4 ± 6.0	9.3 ± 5.5	10.5 ± 6.0

^a^Ethnic difference, Maya versus non-Maya, *p* <  0.05

For all women in the sample, there was a significant correlation between EBV-Ab values and CRP_Plasma_ levels (Spearman ρ = 0.57, *p* < 0.001), but PSS scores were not significantly correlated with either EBV-Ab values (ρ = − 0.08, ns) or CRP_Plasma_ levels (ρ = − 0.02, ns). Similar correlation results among stress measures were obtained when the ethnic groups were considered separately.

Table 2 presents results for bivariate analyses of the relation between stress measures and reported symptoms (none vs. any level of symptom experience) for all participants. The table gives means and standard deviations of the PSS scores, and medians of the CRP_Plasma_ and EBV-Ab levels. For nine of the 12 symptoms, women who reported the symptom had a significantly higher PSS score compared to women who did not report the symptom (*p* ≤ 0.001). Women with hot flashes and headaches had higher PSS scores, with *p*-values of 0.004 and 0.006, respectively – slightly above the conservative Bonferroni correction of *p* ≤ 0.001. EBV-Ab and CRP_Plasma_ values were not significantly higher among women reporting any symptom.

**Table 2:** Bivariate comparisons of stress levels by symptom complaints. Means ± standard deviations for PSS scores or medians for CRP_Plasma_ and EBV-Ab levels shown

Symptom	PSS score by symptom		EBV-Ab value by symptom		CRPPlasma level by symptom	
	No	Yes	No	Yes	No	Yes
Tiredness or lack of energy	13.9 ± 4.2	18.2 ± 5.3***	0.81	1.00*	5.28	5.69
Muscle and joint pain	15.1 ± 4.6	17.9 ± 5.5***	1.00	0.96	5.61	5.64
Nervous or tense	15.3 ± 4.5	18.2 ± 5.5***	0.93	1.00	5.28	5.68
Problems concentrating	15.4 ± 4.5	18.3 ± 5.6***	0.94	0.99	5.57	5.65
Depressed mood or sadness	15.3 ± 4.3	18.4 ± 5.6***	0.93	1.00	4.86	5.72*
Difficulty sleeping	15.1 ± 5.0	18.6 ± 5.2***	0.86	1.01	5.34	5.68
Headaches	16.3 ± 4.7	18.0 ± 5.8**	0.94	1.00	5.48	5.69
Feeling of ants crawling on skin	16.0 ± 4.7	18.3 ± 5.7***	0.82	0.94*	5.33	5.48
Urinary stress incontinence with effort or laughter	16.4 ± 4.9	17.9 ± 5.7*	0.93	0.98	5.34	5.68
Loss of interest in sexual relations	16.3 ± 5.2	18.4 ± 5.4***	1.00	0.96	5.65	5.65
Hot flashes	16.4 ± 5.2	18.2 ± 5.4**	0.94	0.98	5.59	5.67
Night sweats	16.4 ± 5.0	18.5 ± 5.7***	0.84	1.02	5.34	5.68

Two tailed t-tests or Mann-Whitney tests, *p <  0.05; **p <  0.01; ***p ≤ 0.001

The total number of reported symptoms for each individual was significantly correlated with the PSS score (two-tailed Spearman correlations, ρ = 0.42, *p* < 0.001), but not with CRP_Plasma_ (ρ = − 0.02, ns) nor EBV-Ab (ρ = − 0.08, ns) levels. The total symptom intensity score was significantly correlated with the PSS score (ρ = 0.46, *p* < 0.001) and EBV-Ab values (ρ = 0.17, *p* < 0.05).

Table 3 presents the covariates that remained in the models following backwards stepwise logistic regression for each symptom among all participants. PSS score was significantly, positively associated with the report of tiredness, muscle/joint pain, feeling nervous/tense, problems concentrating, depressed mood, difficulty sleeping, and loss of interest in sex after controlling for BMI, SES, education, ethnicity, cohabiting with a husband or partner, parity, smoking, and menopausal status. Odds Ratios for PSS ranged from 1.10 (95% CI 1.04–1.15) for loss of interest in sex to 1.22 (95% CI 1.13–1.32) tiredness. Hot flashes, night sweats, and the feeling of ants crawling on the skin had *p*-values of 0.007, 0.004, and 0.002, respectively – slightly above the conservative Bonferroni correction of *p* ≤ 0.001. EBV-Ab and CRP_Plasma_ levels were not significantly associated with any of the symptoms.

**Table 3:** Logistic regression analyses of symptom reports. 95% Confidence Intervals shown for Odds Ratios

With PSS scores			With CRP_Plasma_			With EBV−Ab		
Variable	Odds Ratio (95% CI)	Signif.	Variable	Odds Ratio (95% CI)	Signif.	Variable	Odds Ratio (95% CI)	Signif.
Tiredness								
PSS Score	1.22 (1.13−1.32)	**< 0.001**	[none remained in model]			Parity	1.41 0.94−2.10	< 0.1
Parity	1.36 (1.02−1.83)	< 0.05						
Muscle and Joint Pain								
PSS Score	1.11 (1.04−1.18)	**< 0.001**	SES	0.80 0.64−1.01	< 0.1	[none remained in model]		
Education	0.95 (0.80−0.95)	< 0.01						
Nervous or Tense								
PSS Score	1.12 (1.06−1.18)	**< 0.001**	Cohabiting (ref)			EBV-Ab	1.76 (1.00−3.12)	= 0.05
			not cohabiting	0.41 (0.19−1.01)	< 0.05			
Parity	1.36 (1.06−1.74)	< 0.05				Cohabiting (ref)		
						not cohabiting	0.39 (0.18−0.85)	< 0.05
BMI	0.96 0.91−1.01	< 0.1						
Problems concentrating								
PSS Score	1.14 (1.08−1.21)	**< 0.001**	Menopause Pre- (Ref)			Non-smoker (ref)		
			Peri-	3.99 (1.31−12.09)	< 0.05	Smoker	3.50 (0.97-12.59)	< 0.1
			Post-	1.57 (0.65−3.80)	ns			
			BMI	1.09 (1.00−1.19)	< 0.05			
Depressed mood								
PSS Score	1.14 (1.07−1.20)	**< 0.001**	BMI	1.10 (1.02−1.18)	< 0.05	BMI	1.09 (1.01−1.18)	< 0.05
BMI	1.07 (1.02−1.13)	= 0.01				Education	0.92 (0.83−1.01)	< 0.1
Difficulty sleeping								
PSS Score	1.18 (1.11−1.25)	**< 0.001**	Education	0.85 (0.76−0.96)	= 0.01	Education	0.91 (0.81−1.01)	< 0.1
Cohabiting (ref)			Menopause Pre- (Ref)			BMI	0.94 (0.87−1.01)	< 0.1
Not cohabiting	2.04 (1.12−3.70)	< 0.05						
			Peri-	1.21 (0.49−2.98)	ns			
			Post-	3.56 (1.37−9.25)	< 0.05			
Ethnicity						Ethnicity		
Maya (ref)						Maya (ref)		
Non-Maya	0.53 (0.30−0.92)	< 0.05				Non-Maya	0.34 (0.16−0.74)	< 0.01
Parity	1.25 (0.97−1.61)	< 0.1						
Headaches								
PSS Score	1.06 (1.01−1.11)	< 0.05	CRP_Plasma_	1.19 (0.97−1.45)	*p*< 0.1	[none remained in model]		
Education	0.93 (0.87−0.99)	< 0.05	Ethnicity Maya (ref)					
			Non−Maya	2.24 (1.07−4.67)	< 0.05			
Cohabiting (ref)								
Not cohabiting	0.63 (0.38−1.05)	< 0.1						
Feeling of ants crawling on skin								
PSS Score	1.08 (1.03−1.13)	= 0.002	CRP_Plasma_	1.22 1.00−1.49	< 0.1	Education	0.90 (0.81–1.00)	= 0.05
			Menopause Pre- (ref)					
			Peri-	2.89 (1.08−7.76)	< 0.05			
			Post-	1.58 (0.69−3.66)	ns			
Stress Incontinence								
PSS Score	1.05 (1.00−1.10)	< 0.05	[none remained in model]			[none remained in model]		
Menopause Pre- (ref)								
Peri-	0.72 (0.38−1.38)	ns						
Post-	0.54 (0.30−0.95)	< 0.05						
Loss of interest in sex								
PSS Score	1.10 (1.04−1.15)	**< 0.001**	Cohabiting (ref)			Cohabiting (ref)		
			not cohabiting	0.30 (0.14−0.64)	= 0.002	not cohabiting	0.48 (0.23–1.01)	= 0.05
Cohabiting (ref)						SES	0.83 (0.69−0.99)	< 0.05
Not cohabiting	0.26 (0.15−0.43)	**< 0.001**						
Ethnicity								
Maya (ref)								
Non-Maya	0.60 (0.36−1.02)	< 0.1						
Hot flashes								
PSS Score	1.07 (1.02−1.12)	= 0.007	[none remained in model]			Cohabiting (ref)		
						not cohabiting	0.54 (0.26–1.13)	= 0.1
Menopause Pre- (ref)						Menopause Pre- (ref)		
Peri-	1.90 (0.99−3.66)	< 0.1				Peri-	2.12 (0.94−4.77)	< 0.1
Post-	1.73 (0.97−3.08)	< 0.1				Post-	2.22 (0.91−5.43)	< 0.1
Parity	1.23 (0.99−1.53)	< 0.1						
Night sweats								
PSS Score	1.07 (1.02−1.13)	= 0.004	CRP_Plasma_	1.26 (1.01−1.57)	< 0.05			
Cohabiting (ref)			Cohabiting (ref)			Cohabiting (ref)		
Not cohabiting	0.40 (0.23−0.69)	**= 0.001**	not cohabiting	0.28 (0.12−0.63)	= 0.002	not cohabiting	0.24 (0.11−0.53)	**< 0.001**
Menopause Pre- (ref)			Menopause Pre- (ref)			Menopause Pre- (ref)		
Peri-	2.06 (1.05−4.06)	< 0.05	Peri-	2.37 (0.92−6.10)	ns	Peri-	3.03 (1.27−7.21)	< 0.05
Post-	2.12 (1.16−3.88)	< 0.05	Post-	2.60 (1.05−6.42)	< 0.05	Post-	2.02 (0.78−5.22)	ns

Variables entered into the logistic models: ethnicity, SES, education, cohabitation status, parity, smoking, body mass index, and menopausal status.  Significance set to *p* < = 0.001

Along with the PSS score, not cohabiting with a husband or partner significantly decreased report of the loss of interest in sex and the likelihood of night sweats. Along with the PSS score, number of children was positively associated with the risk of tiredness and nervousness, although not at the level of *p* ≤ 0.001. Overall, the PSS score was the variable most likely to be associated with symptom frequencies.

When the logistic regressions were carried out separately by ethnicity, for Maya, the PSS score was significantly associated with tiredness, feeling nervous/tense, difficulty concentrating, depressed mood, and night sweats (*p* ≤ 0.001); for non-Maya, the PSS score was significantly associated with reported tiredness, depressed mood, and sleep difficulties (not shown). CRP_Plasma_ levels and EBV-Ab values were not significantly associated with any symptom reports for Maya or non-Maya when ethnic groups were examined separately.

As shown in Table 4, the PSS score was significantly associated with the intensity (*nada = 0 to muchisimo = 3*) of ten of the 12 reported symptoms, including hot flashes and night sweats (*p* ≤ 0.001). CRP_Plasma_ levels and EBV-Ab values were not significantly associated with the intensity of any symptom reports. When regressions were carried out separately by ethnicity, among Maya participants, the PSS score was significantly associated with the intensity of feeling tired, muscle/joint pain, feeling nervous/tense, difficulty concentrating, depressed mood, difficulty sleeping, and night sweats (p ≤ 0.001); for non-Maya, the PSS score was significantly associated with the intensity of the same symptoms except for night sweats. Among the Maya, the association between the PSS score and hot flashes approached significance (*p* = 0.002). CRP_Plasma_ levels were not associated with symptoms among the Maya or non-Maya. EBV-Ab values were not significantly associated with any reported symptom intensity among the Maya or non-Maya.

**Table 4:** Linear regression analyses of symptom reports

With PSS scores				With CRP_Plasma_				With EBV-Ab			
Variable	Beta	t	Signif.	Variable	Beta	t	Signif.	Variable	Beta	t	Signif.
Tiredness											
PSS Score	0.39	6.8	**< 0.001**	CRP_Plasma_	0.19	2.3	< 0.05	Education	−0.20	− 2.3	< 0.05
Education	−.11	2.0	< 0.05								
Muscle and Joint Pain											
PSS Score	0.31	5.4	**< 0.001**	Education	−0.27	−3.2	< 0.01	Education	−0.26	−2.3	< 0.01
Education	−0.22	−3.8	**< 0.001**								
Nervous or Tense											
PSS Score	0.38	6.8	**< 0.001**	CRP_Plasma_	0.17	2.1	< 0.05	EBV-Ab	0.18	2.1	< 0.05
Cohabiting	0.10	1.7	< 0.1	Cohabiting	0.15	1.8	< 0.1	Smoker	0.19	2.3	< 0.05
Smoker	0.16	2.9	< 0.01	Smoker	0.20	2.4	< 0.05				
Difficulty Concentrating											
PSS Score	0.33	5.6	**< 0.001**	BMI	0.23	2.7	< 0.01	[No variables left in model]			
Parity	0.12	2.1	< 0.05	Parity	0.14	1.7	< 0.1				
Depressed mood											
PSS Score	0.51	9.7	**< 0.001**	CRP_Plasma_	0.19	2.3	< 0.05	Education	−0.18	−2.1	< 0.05
Parity	0.13	2.5	< 0.05	Education	−0.18	−2.2	< 0.05				
				Parity	0.18	2.1	< 0.05				
Difficulty sleeping											
PSS Score	0.39	6.9	**< 0.001**	Menopause status	0.27	3.3	**< 0.001**	Education	−0.22	−2.6	< 0.05
Menopause status	0.13	2.2	< 0.05	Education	−0.21	−2.6	< 0.05				
Head aches											
PSS Score	0.26	4.5	**< 0.001**	Ethnicity	−0.19	−2.2	< 0.05	[No variables left in model]			
Cohabiting	0.12	2.0	< 0.05	Smoker	−0.16	−1.8	< 0.1				
Smoker	−0.12	−2.0	< 0.1								
Feeling of Ants Crawling on the Skin											
PSS Score	0.24	3.9	**< 0.001**	CRP_Plasma_	0.21	2.4	< 0.05	Education	−0.30	−3.7	**< 0.001**
SES	−0.10	−1.7	< 0.1	BMI	−0.18	−2.0	< 0.05				
Menopause status	0.10	1.7	< 0.1								
Stress Incontinence											
PSS Score	0.15	2.4	< 0.05	Cohabiting	0.16	1.9	< 0.1	BMI	0.24	2.9	< 0.01
BMI	0.13	2.1	< 0.05					Cohabiting	0.18	2.1	< 0.05
Cohabiting	0.11	1.8	< 0.1					Menopause status	−0.14	−1.7	< 0.1
Loss of interest in sex											
PSS Score	0.18	2.9	< 0.01	Cohabiting	0.31	3.7	**< 0.001**	Cohabiting	0.22	2.6	< 0.05
Education	−0.11	−1.8	< 0.1					SES	−0.18	−2.1	< 0.05
Cohabiting	0.27	4.6	**< 0.001**								
Menopause status	0.14	2.4	< 0.05								
Ethnicity	0.10	1.7	< 0.1								
Hot flashes											
PSS Score	0.23	3.8	**< 0.001**	[No variables left in model]				SES	−0.19	−2.3	< 0.05
Menopause status	0.13	2.2	< 0.05					BMI	0.17	2.0	< 0.05
BMI	0.15	2.6	< 0.01					Menopause status	0.15	1.8	< 0.1
Night sweats											
PSS Score	0.24	4.2	**< 0.001**	CRP_Plasma_	0.19	2.4	< 0.05	Menopause status	0.23	2.8	< 0.01
Menopause status	0.22	3.7	**< 0.001**	Menopause status	0.30	3.7	**< 0.001**	Education	−0.21	−2.7	< 0.01
Parity	0.15	2.5	< 0.05	Education	−0.22	− 2.7	< 0.01	Cohabiting	0.28	3.4	**< 0.001**
Cohabiting	0.12	2.1	< 0.05	Cohabiting	0.22	2.7	< 0.01				

Variables entered into the linear models: ethnicity, SES, education, cohabitation status, parity, smoking, body mass index, and menopausal status. Significance set to *p* < = 0.001

Along with the PSS score, level of education was negatively associated with the intensity of muscle and joint pain. Cohabiting with a husband or partner was positively associated with the intensity of the loss of interest in sex. Progression through menopause was associated with the increased intensity of night sweats.

## Discussion

In this urban population of women aged 40 to 60 from Campeche, Mexico, hot flashes and night sweats were not the most commonly reported symptoms. This finding is consistent with other studies that have found aches and stiffness [32, 60, 65], lack of energy [59], and tiredness or fatigue [61, 66] to be more common than hot flashes.

Correlations between self-report measures and biological markers of stress tend to be small or moderate [38]. In the case of this Campeche sample, there were no significant relationships between self-reports of stress (PSS score) and the potentially stress-sensitive biological measures (EBV-Ab and CRP levels), although the two measures of immune function were positively and significantly correlated. None of the measures used in this study were solely measuring stress; there are many factors that can influence immune system activity, and the PSS measures perceptions of stress which can be quite variable in different individuals [38]. It may be that biological measures were elevated in relation to immunological stress, but that immunological activity did not correlate with the impact of stress on the participants within the context of their lives. It may be that these particular biomarkers were not sensitive enough, or that the biomarkers could not effectively measure stress as perceived by the person.

Self-reported PSS was found to be significantly associated with nine of the most common symptoms in bivariate analyses, and with seven symptoms after controlling for potential covariates, whereas neither CRP nor EBV-Ab were associated with symptoms. PSS scores were also associated with the intensity of ten reported symptoms, including hot flashes and night sweats. No other variable in the logistic or linear models was associated with so many midlife symptoms. Our findings are similar to the relationship reported by SWAN researchers who found that the PSS score was significantly associated with vasomotor symptoms [15].

When logistic regressions were carried out separately by ethnicity, the PSS score was significantly associated with five of the reported symptoms among Maya women, including night sweats (*n* = 144). However, among non-Maya women (*n* = 121) the PSS score was significantly associated with only three of the symptoms. These ethnic differences may reflect cultural differences in either the experience or reporting of vasomotor and other symptoms [38, 67]. For Maya women, symptoms may be a means of expressing feelings of stress [11], more so than for non-Maya women.

In results reported here, Maya and non-Maya women did not differ in mean PSS scores; however, Maya women with higher PSS scores were more likely to report a higher intensity of hot flashes (*p* = 0.002) and the presence of and a higher intensity of night sweats (*p* ≤ 0.001). It is of interest to note that earlier literature found an absence of hot flash report among Maya women in the Yucatán peninsula [42, 43, 68]. In contrast, the study presented here did not find an ethnic difference in hot flash report, but instead found a greater likelihood of vasomotor symptoms among the Maya in relation to higher perceived levels of stress. This bears further investigation.

This study provides only modest support for the idea that immune biomarkers applied as stress-sensitive measures are associated with the frequency of symptoms at midlife. The relationship between CRP_Plasma_ levels and the occurrence of depressed mood did not reach significance, although there was a suggestion of a relationship in bivariate and linear regression analyses (*p* < 0.05). The association between CRP levels and depressed mood has been previously noted [69].

In agreement with our findings, one other previous study did not find a relationship between CRP and hot flashes [70]. In SWAN, women who had a higher frequency of hot flashes had significantly higher levels of CRP and other biological markers of inflammation, but there was no significant association between night sweat frequency and inflammatory markers [71].

EBV-Ab values were not significantly associated with hot flashes or night sweats in terms of yes/no frequency or intensity of report. To our knowledge, this is the first study to examine symptoms at midlife in relation to levels of EBV-Ab. Although CRP_Plasma_ and EBV-Ab have been used as stress-sensitive biological measures in the study of stress in the past [47, 50, 51], in this study neither CRP_Plasma_ nor EBV-Ab levels were associated with symptoms at midlife to the same extent as the self-reported stress measure, PSS.

Self-reports of stress have been associated with the frequency of hot flashes, both with short-term reports such as hassles scales [72] and reports of chronic stress [73]. However, there may well be differences in the association between stress and hot flashes depending upon the manner in which hot flashes are measured. For example, in a prospective study of mood and hot flashes, negative mood was associated with fewer objectively measured hot flashes but was associated with more frequent self-reported hot flashes [19]. In this study, PSS scores were significantly associated with the intensity of vasomotor symptoms when correction for multiple testing was applied, and PSS scores tended to be associated with the likelihood of vasomotor symptoms (*p* = 0.007 and *p* = 0.004 for hot flashes and night sweats), unlike the physiological measures of stress.

In general, women who reported high levels of perceived stress were also more likely to report a broad array of symptoms. Some of these symptoms are specific to menopause, such as night sweats, but many are more general concerns of men and women of a broad age range. These symptoms are associated with multiple factors. For example, not cohabiting with a husband or partner significantly decreased report of the loss of interest in sex.

Self-reported stress is clearly implicated as associated with symptoms, especially among the Maya in this sample. It is, however, unclear to what degree stress may be a causal factor in inducing these symptoms, or if instead the symptoms are a causal factor in the stress levels. There could be a reciprocal effect, with stress inducing symptoms that in turn lead to greater perceptions of stress. Few women reported either no symptoms (1.4%) or all 12 symptoms (5.4%), suggesting that there is not a simple relation between being under stress and having all symptoms; different women suffer from different symptoms, and these are likely to differ in the importance of stress levels for their occurrence. It is unclear why the stronger association between perceived stress and vasomotor symptoms is present among Maya but not non-Maya participants. The women may differ in beliefs about how stress should be reported, since ethnic differences in self-reports of stress are found in other populations [38].

This study has limitations. While the sample is likely to broadly represent the population of women at mid-life in Campeche due to the multiple strategies used for contacting potential participants, it is not a random sample. The sample size is small, with 305 women providing PSS scores, and only 162 and 157 women with EBV-Ab and CRP measures, respectively. We did not find the expected relationship between the PSS and the two biomarkers. Also, this paper has relied upon self-reports of hot flashes and night sweats as well as other symptoms. As noted, a previous study has shown a difference in the relation between stress and either subjectively reported or objectively measured hot flashes [19]. Finally, this study is cross-sectional and, therefore, cannot derive causation from associations between the variables used in analyses.

## Conclusions

In support of our primary hypothesis, perceived stress was associated with the intensity of hot flashes and night sweats. In logistic and linear regressions, perceived stress was the variable most consistently associated with each of the 12 symptoms studied. This was not true for the potentially stress-sensitive biological measures of EBV-Ab or CRP_Plasma_. There were ethnic differences in the associations between measures of stress and symptom frequency and intensity. Maya women demonstrated a relationship between perceived stress and five symptoms, including night sweats, while the non-Maya demonstrated no association between between perceived stress and vasomotor symptoms, suggesting that either symptoms or stress were experienced and/or reported in culture-specific ways.
